# Phenological Model to Predict Budbreak and Flowering Dates of Four *Vitis vinifera* L. Cultivars Cultivated in DO. Ribeiro (North-West Spain)

**DOI:** 10.3390/plants10030502

**Published:** 2021-03-08

**Authors:** Alba Piña-Rey, Helena Ribeiro, María Fernández-González, Ilda Abreu, F. Javier Rodríguez-Rajo

**Affiliations:** 1 Sciences Faculty of Ourense, Campus da Auga, University of Vigo, 32004 Ourense, Spain; apina@uvigo.es; 2 Earth Sciences Institute (ICT), Pole of the Faculty of Sciences, University of Porto, 4169-007 Porto, Portugal; helena.ribeiro@fc.up.pt (H.R.); ianoronh@fc.up.pt (I.A.); 3 Department of Geosciences, Environment and Spatial Plannings of the Faculty of Sciences, University of Porto, 4169-007 Porto, Portugal; 4 Department of Biology, Faculty of Sciences, University of Porto, 4169-007 Porto, Portugal

**Keywords:** phenological modeling platform, budburst, flowering, prediction, GDD model, GDD triangular model, uniforc model

## Abstract

The aim of this study was to assess the thermal requirements of the most important grapevine varieties in northwestern Spain to better understand the impact of climate change on their phenology. Different phenological models (GDD, GDD Triangular and UniFORC) were tested and validated to predict budburst and flowering dates of grapevines at the variety level using phenological observations collected from Treixadura, Godello, Loureira and Albariño between 2008 and 2019. The same modeling framework was assessed to obtain the most suitable model for this region. The parametrization of the models was carried out with the Phenological Modeling Platform (PMP) platform by means of an iterative optimization process. Phenological data for all four varieties were used to determine the best-fitted parameters for each variety and model type that best predicted budburst and flowering dates. A model calibration phase was conducted using each variety dataset independently, where the intermediate-fitted parameters for each model formulation were freely-adjusted. Afterwards, the parameter set combination of the model providing the highest performance for each variety was externally validated with the dataset of the other three varieties, which allowed us to establish one overall unique model for budburst and flowering for all varieties. Finally, the performance of this model was compared with the attained one while considering all varieties in one dataset (12 years × 4 varieties giving a total number of observations of 48). For both phenological stages, the results showed no considerable differences between the GDD and Triangular GDD models. The best parameters selected were those provided by the Treixadura GDD model for budburst (day of the year (t_0_) = 49 and base temperature (Tb) = 5) and those corresponding to the Godello model (t_0_ = 52 and Tb = 6) for flowering. The modeling approach employed allowed obtaining a global prediction model that can adequately predict budburst and flowering dates for all varieties.

## 1. Introduction

In the near future, changes in local and regional atmospheric patterns are expected due to climate variations which could significantly affect grapevine phenology, grape production and wine quality [1,2]. Temperature is the main variable driving changes in grape phenology [3,4,5,6], together with photoperiod [7,8,9,10] and precipitation [11,12,13]. *Vitis vinifera* is very sensitive to temperature variations, requiring a narrow average temperature of 12 to 22 °C during the growth period for a successful harvest [14,15]. Therefore, the phenology evolution of the different varieties can be used as a bioindicator of changes associated with global warming [16,17,18,19]. For example, in California, Nemani et al. [20] found an increase in crop quality and yield over the last 50 years that was related to a decrease in the number of frosts, an earlier onset of growth during the spring and a longer growing season influenced by unequal warming. Alterations in the physiological development and growth of grapevine have also been documented [8,21]. Therefore, climate change is expected to influence the close relationship between grapevine development and climate, affecting the yield and quality of the final products of this crop [11]. Many studies at the regional and global level have evaluated how the increase in air temperature over the last century has influenced the main agricultural ecosystems [1,7,22] as well as the assessment of future trends and impacts [3,23]. In the 20th century, the temperature increased between 0.5 and 1 °C on average, possibly due to the CO_2_ concentration rise from 280 ppm to 400 ppm [24,25]. As the trend of the CO_2_ concentration increases in the atmosphere is expected to continue, it is probable that the global average temperature will rise by 0.2 to 0.3 °C per decade [26]. At the end of the 21st century, the temperature rise could exceed 1.5 °C [25].

Therefore, it is essential to know how temperature influences both the reproductive cycle and vegetative development, and to observe differences between vegetation varieties [27,28]. Variations of this meteorological factor affect cell metabolism, the level of carbon accumulation and other biochemical processes [29,30]. This information is essential to identify which varieties are more suitable for the evolving climate conditions expected in a given area. At the phenophase level, temperature plays a decisive role in the appearance of shoots during the BBCH phenophases 03 (End of bud swelling: buds swollen, but not green), 05 (“Wool stage”: brown wool clearly visible), 07 (Beginning of bud burst: green shoot tips just visible) and 09 (Bud burst: green shoot tips clearly visible) [31,32,33]. At the beginning of the dormancy, cold temperatures are needed, while towards the end of this period, warm temperatures are required for optimal budburst [34,35]. In the case of flowering, temperatures and degrees-day of growth are decisive [36,37].

The advance or delay in the beginning of the phenological phases accompanied by the shortening of their duration has been highly correlated with the observed increases in temperature [3,7,22,38]. In some European countries, several advances in phenological events have been recorded [3,39] ranging from 6 to 25 days, with an average of 3 to 6 days for each temperature degree increased over the last 30–50 years during the growing seasons [38]. The consequences of these changes could influence the speed of plant growth and the amount and composition of several grape components such as sugar, flavor modification and acidity variations [40,41,42,43]. Night temperatures below 15 °C increase acidity, while temperatures between 5 °C and 15 °C favor the concentration of aromas [44].

Agrometeorological models represent a suitable tool for the study of the climate variations in the grapevine, as their phenological development is spatially regulated by the temperature [45,46,47,48]. These kinds of models are based on the accumulated heat sum of degree-days, taking into account a minimum threshold temperature from a defined date until the appearance of a certain phenological phase [9,27,31,49]. The correct choice of the starting date of heat accumulation, and mainly the threshold temperature, will condition the predictive aptitude of the statistical model [49,50]. In the specific case of grapevines, different threshold temperatures have been proposed, from 10 °C [45] to temperatures below 4 °C [51,52]. In phenological modeling, unified models are often developed on a multi-regional scale [27]. However, it is also important to develop site-specific models, since plants can adapt to their environment showing variations in their phenological developmental stages [10,27,41,53]. Among other applications, the information provided by this kind of models supports the knowledge for the adaptation capacity of a given variety to different climate zones, or detect behavior discrepancies of different varieties in the same region. Also, to improve and to optimize several viticultural aspects as harvest planning, integrated pest and disease management, viticultural practices and the organization of work in the winery. Concerning climate change, if phenological models are applied to future climate change scenarios, it is possible to project upcoming impacts on the grapevine industry [54].

Our study sought to assess and validate temperature-based phenological models to predict the date of budburst and flowering of four grape varieties in the Ribeiro Designation of Origin (Ribeiro DO), one of Spain’s main wine-growing areas near the Eurosiberian and Mediterranean areas. The best performing models were selected in terms of efficiency, as they are intended to be applied as a useful tool for optimizing agricultural practices and mitigating the expected climate change impacts on *Vitis vinifera* varieties. 

## 2. Results

**Figure 1:**
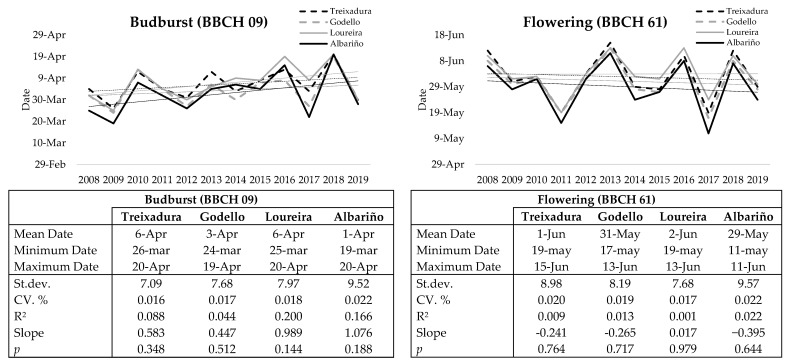
Phenological dates of budburst and flowering of the 4 cultivars (Treixadura, Godello, Loureira and Albariño) during 2008–2019 (Figures). Average, maximum and minimum budburst and flowering start date, standard deviation and standard deviation in percentage (Tables).

The opening of the buds (BBCH 09) took place at the beginning of April with only 6 days of difference on average between the different varieties. The beginning of flowering (BBCH 61) occurred between the last days of May and the first days of June. On average, an amount of 5 days of difference between the varieties was observed, with a slight downward trend causing a small advance at the beginning of this phase, being more remarkable in the Albariño variety. In general, Albariño was the earliest variety while Loureira was the latest in both cases (Figure 1). 

Temperature-based phenological models were estimated for budburst and flowering grapevine phenophases using different beginning dates of the meteorological dataset: 1 September of the previous year, and 1 January of the current year. It was observed that the model optimization converged to the same region of the parameter space. The differences found between both dates taking into account the efficiency (EFF) within and between each type of model (except for the UniForc model), were very low. However, the data set beginning on the 1st of January allowed achieving models with a slightly higher EFF and lower root mean square error (RMSE)(data not shown). So, this dataset was used in the subsequent model calibration.

Comparing the estimated models for budburst, scarcely differences were detected in terms of efficiency between the GDD model and the Triangular GDD. Among both models, the EFF oscillated between 0.954–0.810 and the RMSE between 3.809–1.890 for the four varieties. However, the GDD model presented lower oscillation in the range of the fitted parameters (except for the Loureira variety). In general, the earlier t0 was set for the Godello and Treixadura varieties (t0 52 and 49 respectively) corresponding to late February. The latest was detected for Loureira, corresponding to the end of March. 

It was observed that the best-fitted parameters converged in the following values for each variety (Table 1).

For the GDD model, Tb values were 8, 5, 10 and 5 °C in Albariño, Godello, Loureira and Treixadura varieties, respectively.

In the GDD Triangular model, minT values were 9, 5, 10 and 7 °C, Tb values were 13, 16, 14 and 23 °C and maxT values were 24, 50, 29 and 26 °C in Albariño, Godello, Loureira and Treixadura varieties, respectively.

Following the UniForc model, the values obtained for the d parameter were −19.326, −0.265, −40.000 and −0.923, whereas for the e parameter the values obtained were 11.538, 15.507, 12.164 and 10.811 °C in the Albariño, Godello, Loureira and Treixadura varieties, respectively.

The phenological models estimated for flowering recorded very similar results in terms of efficiency (EFF between 0.966–0.900 and an RMSE of 2.768–1.474). The GDD Triangular model showed a slightly higher adjustment in three of the four varieties. In this case, the Albariño variety was the first to initiate the accumulation of heat for flowering, starting in late January. Loureira again was the latest variety, starting in mid-March. The best-fitted parameters estimated for each variety for the different models were:

The GDD model, where the values of Tb were 7 °C for the Albariño and Treixadura varieties, and 6 °C for Godello and Loureira.

The GDD Triangular model, where the minT values were 7, 6, 6 and 7 °C, the values of Tb 27, 23, 26 and 24 °C, and for the maxT 32, 31, 32 and 25 °C in the Albariño, Godello, Loureira and Treixadura varieties, respectively.

The UniForc model, where the values of d were −0.937, −0.217, −0.293 and −0.281, and for e were 12.772, 19.490, 14.429 and 17.393 °C.

For both phenological stages, the results did not register considerable differences between the GDD and the Triangular GDD models. It is important to highlight that the GDD model has less complexity for its practical application and higher degrees-of-freedom. Therefore, the optimized GDD phenological model for the prediction of the budburst and flowering dates was chosen as the best-performing model. Their parameters for each variety can be observed in Table 2. These models with fixed parameters were externally validated with the dataset of the other grapevine varieties aiming to find an overall model that can best predict the date of budburst and flowering. The more accurate model for predicting the budburst date used the optimal values of the Treixadura model, which achieved the higher EFF and lower RMSE for every other grape varieties. In the case of flowering, the Godello model was the best predictor for this phenophase with the highest EFF and lowest RMSE for all study varieties (Table 3). 

**Figure 2:**
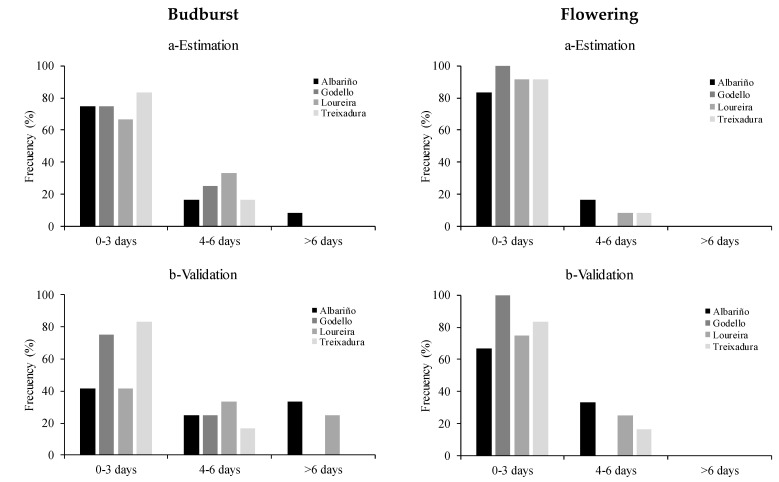
Frequency in the percentage of difference in days between predicted and observed budburst and flowering dates for grapevine varieties, **a**- used in the GDD model estimation by budburst and flowering **b**- and for GDD validation using the best-fitted model (Treixadura for budburst and Godello for flowering) with the other grapevine varieties.

Figure 2 shows the differences in the number of days between the predicted and observed budburst and flowering dates, plotted against the frequency of occurrence in three classes of days [0–3], [3–6] and [>6] in the estimation and external validation. In the estimation phase, differences below 3 days were observed in about three-quarters of the cases for budburst. Only one of the cases in the Albariño variety showed a difference of more than 6 days. For flowering, almost 90% of the cases registered differences of less than 3 days (Figure 2a). In the validation phase, using the GDD model estimated for budburst in Treixadura (t0 49 DOY and Tb 5 °C) the differences between the predicted and observed values were less than 3 days in 60% of the cases. Albariño and Loureira were the only varieties that presented cases with more than 6 days of difference. For the flowering phase, using the GDD model estimated for Godello (t0 52 DOY and Tb 6 °C), 81% of the cases presented differences between the predicted and observed values in a range lower than 3 days, whereas the remaining 18% presented differences between 4 and 6 days (Figure 2b).

**Figure 3:**
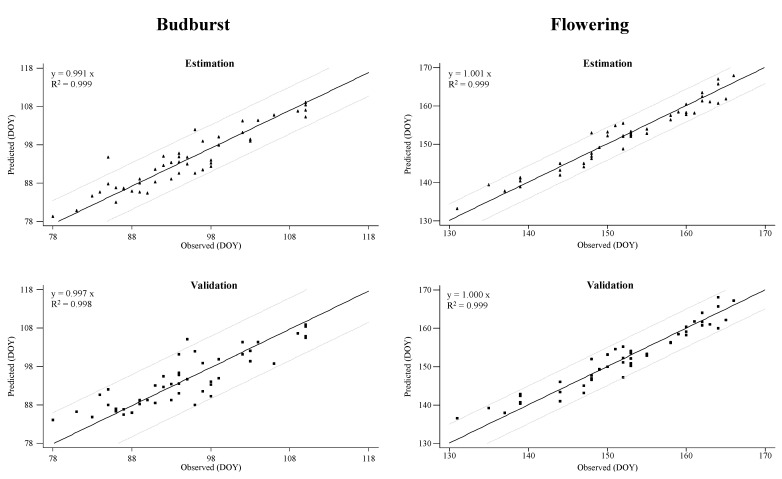
Predicted and observed dates using the Treixadura GDD model for budburst stage and Godello GDD model for the flowering stage. Closed triangles (▴) represent data used for the model estimation and closed square (▪) represent data used for the model validation. A 95% Confidence interval is represented by the gray dashed line.

The results of the linear regressions across the origin between the predicted and observed budburst/flowering dates (respectively), from the Treixadura GDD model for the budburst stage and the Godello GDD model for the flowering stage, are shown in Figure 3. In general, the regression coefficients (b0) are close to 1.0, both in the estimation and in the validation steps, indicating the absence of bias (Figure 3).

**Figure 4:**
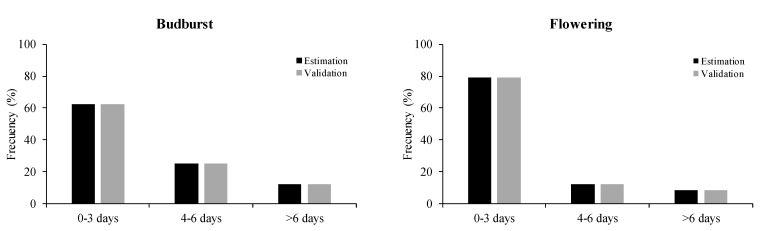
Frequency in the percentage of difference in days between predicted and observed budburst and flowering dates for the GDD Global model (estimation and validation values).

The global GDD model estimated the data of the four varieties altogether to predict budburst and flowering; the best-fitted parameters were t0 = 63 and 52, respectively, and a base temperature of 7 °C for both budburst and flowering. The most accurate model, with the lowest RMSE and higher efficiency, was obtained for the flowering stage (Table 4). The difference in days between the predicted and observed budburst and flowering start dates for this model showed that 62% of the cases for budburst and 79% of the cases for flowering presented differences of less than 3 days (Figure 4). 

## 3. Discussion

In recent years, phenology has been considered as a key to many studies on climate change, mainly due to the effects of temperature on the life cycles of plants, especially on the grapevine. Several authors have reported an increasing temperature trend in certain wine-producing areas [55,56]. In northeastern Spain, Ramos et al. [57] found general warming of 1.0 to 2.2 °C during the period of vegetation. Another study in northwestern Spain showed a significant trend towards an increase in the temperature-related bioclimatic indices [58]. Consequently, changes in the annual growth cycle and phenological stages of the vine were already being observed [13,59,60]. When we analyzed the phenology of the four grapevine varieties representatives of the Ribeiro DO (Ribeiro Designation of Origin) over 12 years, an oscillation was observed in the variability of the timing, mainly a small delay in the budburst stage start date. Studies conducted in the Alsace region pointed out that the inter-annual variability of budbreak decreased in the period 1989–2015 [61]. These findings were shared in different vineyards around the world [11,62]. Budburst is one of the most important stages of development, as it represents the start of vegetative growth. An increase in temperature can bring advances or delays in its start date, inducing important consequences in the following reproductive stages [63]. During winter, the grapevine enters into a dormant state to overcome adverse conditions by means of interruption of the growth [35]. To overcome this stage, the plant must accumulate a certain number of chilling units during the first part of the dormancy, while the vine needs warm temperatures in the last part to stimulate a rapid and homogeneous budburst [34]. Moreover, the flowering stage showed a predisposition to advance their start date, recording certain variations in these dates. During recent years (during the period 1989–2015), the inter-annual variability of flowering increased in different regions such as the Alsace region [61], Burgundy region [62] and in other vineyards around the world [1]. Several studies that combine phenological models with climate change scenarios showed that this stage will change in the future, with earlier growth expected in the northern vineyards than in those in southern Europe [64], or in different areas of France [48,65,66,67] and in the Trentino area (Italian-Alps) [41]. The magnitude of the advances was assessed as being between 8 and 10 days until the end of 2100 for flowering [60]. The collection of phenological data from such crucial stages, as budburst and flowering, is important as an indicator of the evolution of the crop.

On the other hand, three temperature-based phenological models were estimated and validated to predict the timing of budburst and flowering, using the phenological dataset of four grapevine varieties (Albariño, Godello, Loureira and Treixadura). Through an iterative process, the parameterization of the models was achieved. The thermal models used in this study were based on the occurrence of a certain phenological stage through a sum of temperatures, starting on a predefined date. The starting date of January 1st of the current year showed a small improvement in efficiency, according to some authors that suggest a start date of the thermal accumulation during the first days of the year [46,68]. Leolini and collaborators [63] revealed that January 1st seems to be more suitable for assessing the budburst date of the vine varieties collected in Southern Europe. Our study showed the initial date for the thermal sum of the budburst stage occurred in late winter (February and March). In general, Godello (t0 = 49–49-34 DOY in GDD, GDD Triangular, UniForc models) and Treixadura (t0 = 49–52-72 DOY in GDD, GDD Triangular, UniForc models) were the earliest varieties, and Albariño (t0 = 62–70-70 DOY in GDD, GDD Triangular, UniForc models) and Loureira (t0 = 79–79-80 DOY in GDD, GDD Triangular, UniForc models) were the latest varieties. These results are in accordance with the data pointed out by several authors in France, Italy, Switzerland and Greece (t0 = 60 DOY) [27], in California (t0 = 51 DOY) [45], in France (t0 = 46 DOY) [48], or at the northern boundary of the commercial grapevine production areas in Europe (average t0 = 60 DOY) [49]. Regarding the flowering stage, Albariño was the first variety to initiate the heat accumulation with t0 = 27–27-11 DOY, which was noted after considering the three models. Godello and Treixadura varieties recorded the same t0 = 52 DOY for the GDD and GDD Triangular models and t0 = 28 DOY or t0 = 26 DOY for the UniFORC model, respectively. The latest variety is Loureira with t0 = 72–70-70 DOY. These results are slightly different from those indicated in two wine-growing areas of Portugal, in which the third part of March was noted as the most accurate starting date for the heat accumulation calculation for the flowering stage [10]. Some research carried out in the most important wine-growing areas in France, Switzerland and Italy noted that the best starting date for heat accumulation units was March 1st [27]. This variability may be because flower formation in the grapevine is a very complex process that is strongly influenced by the environment and vine growing practices [69]. The transcendental stages in the flower formation process can be induction, initiation and early differentiation during season one, and differentiation at budburst during season two [69]. The vine cycle of reproductive development is fulfilled during two successive growing seasons separated by a latency period [70]. 

Afterwards, we sought to determine for each type of model (GDD, GDD Triangular and UniFORC) and variety the estimates of fixed parameters that best predict the dates of budburst and flowering. The best base temperature (Tb) for the GDD model was established to be between 5 to 10 °C for budburst. In the GDD Triangular model, the minimum temperature ranged between 5 to 10 °C, the base temperature was fixed from 13 to 23 °C and the maximum temperature was fixed from 24 to 50 °C. In the case of the flowering stage, the best base temperature for the GDD model was established to be between 6 to 7 °C. In the GDD Triangular model, the minimum temperature was 6 to 7 °C, the base temperature was fixed from 23 to 27 °C and the maximum temperature was fixed from 25 to 32 °C for the four studied varieties. Different authors proposed several base temperatures as the optimum threshold above which the growth level is efficient. For *Vitis vinifera*, 10 °C has been pointed out as the base/minimum temperature [5,46,71,72]. Also, a study carried out in Europe to calculate the grapevine budbreak date on eight varieties noted that for the GDD model, the best base temperature ranged from 0 to 10 °C [63]. Otherwise, the range of base temperatures suitable for budbreak was stated to be from 0.4 °C to 4.6 °C [52]. However, different responses to growth and development after budbreak were found within a temperature range between 15 °C and 35 °C [73]. This base temperature can vary depending on the phenological stage or the studied area. A model for the vineyard flowering carried out in France, Italy, Switzerland, and Greece pointed out 0 °C as the best base temperature [27]. In the Portugal region, a base temperature of 9.2 °C was observed for the Fernão-Pires variety and 8.9 °C for the Castelão variety regarding budburst, flowering, and veraison [74]. On the other hand, the optimal range of temperatures to carry out the photosynthesis process ranged from 25 to 35 °C [75]. Our study showed that the base temperature for budbreak is only close to this optimum in the case of Treixadura, with 23 °C. However, during the flowering process, Albariño and Loureira reached 27 and 26 °C, respectively, while Godello and Treixadura were very close to the optimum (23 and 24 °C, respectively). These base temperature variations can be explained by the phenotypic plasticity of the vine, whose values are influenced by intrinsic plant factors and the local environment [76]. That is, although the varieties are usually well adapted to the specific climatic conditions of the sites where they traditionally grow [9,77], they differ in the thermal accumulation required for a given phenological stage [78,79].

From a statistical point of view, the three model types (GDD, GDD Triangular and UniFORC) used for the prediction of the budbreak and flowering dates showed comparable predictive accuracy. However, under equal predictive capabilities, simpler models should be selected [80]. In this case, the GDD model is the simplest model between the assessed results and obtaining a slightly better EFF and RMSE. 

Our study developed specific phenological models that are particularly suitable for each selected vine variety. The obtained results provide a key tool for winegrowers, since they offer valuable information for vineyards management and planning [81]. Besides, the accurate prediction of phenological stages encourages better practices and timely action to improve vine yields and grape quality [82,83,84,85]. However, climate change is expected to affect wine production worldwide and it is necessary to get a global model to see how the plants will react. The development of both global and regional models is important due to the ecological plasticity of the vine that is usually explored in regions located under the influence of Mediterranean climate where meteorological variables such as temperature or relative humidity showed significant fluctuations in recent years. Therefore, it is important to obtain regional forecasting models to evaluate the effect of climate change more assertively in the bioclimatic conditions of a given wine region. In the present study, a specific model has been obtained for each variety. However, we managed to estimate an accurate model for all varieties, although with a small but acceptable loss in forecasting power, given the possibility of having a global model for our whole study region. Considering this condition, the modeling approach used in our study allowed us to achieve a global prediction model for the 4 varieties that can adequately predict the date of budburst and flowering: EFF = 0.731 and RMSE = 4.153 for budburst, and EFF = 0.881 and RMSE = 2.979 for flowering. The future challenge will be to test this model in more varieties.

In general, our results showed that thermal-time phenological models work better to predict flowering than budburst. This fact may be due to latency and other physiological processes that could have a greater impact on budbreak [60]. Also, there may be some variability in the data collected visually despite the application of standardized criteria, and winter pruning may affect the variability of the stage [80,86,87,88].

## 4. Materials and Methods

### 4.1. Study Area

**Figure 5:**
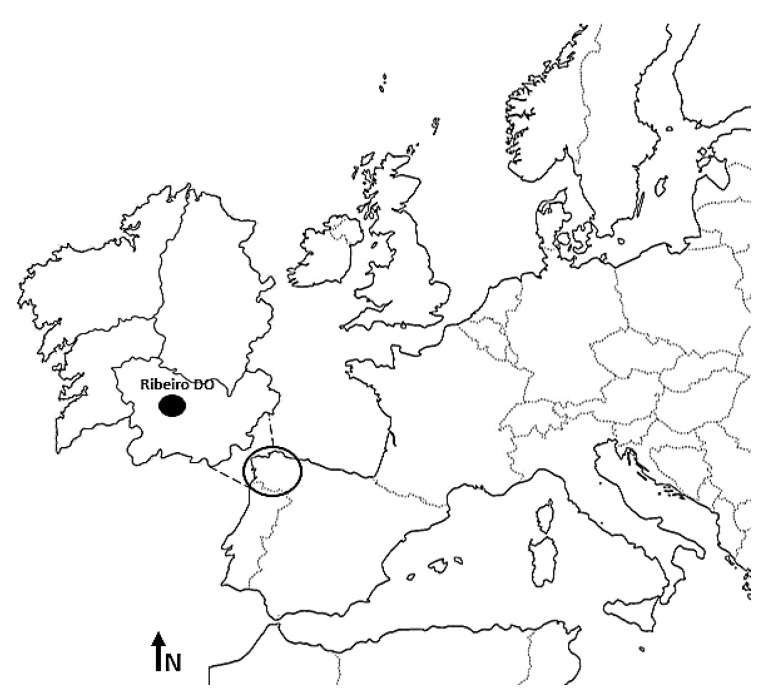
Location of the study area.

The present study was carried out in a vineyard located in the North-West of Spain at 140 m above sea level (42°18′ N, 8°6′ W) belonging to the Ribeiro Designation of Origin (Ribeiro DO) (Figure 5). Following the Multicriteria Climatic Classification (MCC) system, most winemaking areas in this region, watered by the Miño River, would be defined as temperate, warm and sub-humid with very cold nights [89]. Also, the area presents a granitic soil with abundant stones and gravel, as well as a sandy texture with an average depth of between 70 and 100 cm [90].

Meteorological data were obtained from an automatic weather station correlated to a data logger called HOBO Micro Station (Onset, USA), located at the central part of the vineyard (42°19’54´´ N/8°7´34´´ W). The monitored daily parameters were maximum temperature, average temperature, and minimum temperature.

The current study is based on grapevine phenological observations from 2008 to 2019 collected at four plots in the same vineyard with the following separate varieties: Treixadura, Godello, Loureira and Albariño. The observations were conducted in each variety every 6–7 days, increasing to every 3–4 days during the flowering stage. A total of 20 plants per each studied variety were selected for the phenological study using the scale recommended by Lorenz et al. [91], adopted as the standardized scale for phenological grapevine observations by BBCH [92]. A given phenological stage was reached when the event took place in the 50% of the sampled plants for each grape variety at the stages according to the BBCH scale [92]: “09 Opening of buds: leaf tips clearly visible”, “61 Beginning of flowering: 10% of flower hoods fallen”.

### 4.2. Phenological Models

Temperature-based phenological models were tested centered on the proportional relationship between the action of forcing temperatures (*x_t_* corresponding to the daily mean temperature) and the daily rate of phenophase development (denoted by R*f*). So, it was modeled that a specific phenophase occurred at day D*_t_* after a critical value of thermal accumulation was reached (expressed in degree-days or forcing units denoted by F*) from an arbitrary onset date (*t_0_*) (Equation (1)) [93].

(1) $$Phenophase\;\left(D_{t}\right)=\;\sum_{t_{0}}^{D_{t}}Rf(x_{t})\geq F^{*}$$

The daily rate of development (or daily rate of forcing) R*f*, which is a function of temperature, was tested in three different mathematical formulations:

1. Growing Degree Days model (GDD) Equation (2): The GDD model includes three parameters, *t_0_*, *T_b_* and F*. The summation of daily average temperature (*x_t_*) above a specific threshold (*T_b_*) from a specific date (*t_0_*) until an optimum thermal accumulation (F*) was considered [94].
  (2) $$Rf=GDD\left(x_{t}\right)=\{\begin{array}{c} o\;if\;x_{t}<Tb\\ x_{t}-Tb\;ifx_{t}\geq Tb \end{array}\;\;\;$$
2. Growing Degree Days Triangular model Equation (3): The GDD Triangular model represents a non-linear triangular function based on cardinal temperatures, hence the F^*^ takes values from 0 to 1. Four parameters were included for the estimation: *t_0_, T_min_, T_opt_, T_max_* and F^*^.
  (3) $$Rf\left(x_{t}\right)=\{\begin{array}{c} 0\;if\;x_{t}\ll Tmin\\ \frac{x_{t}-Tmin}{Topt-Tmin}if\;Tmin<x_{t}\ll Topt\\ \frac{x_{t}-Tmax}{Topt-Tmax}if\;Topt<x_{t}<Tmax\\ 0\;if\;x_{t}\gg Tmax \end{array}$$
3. UniFORC model Equation (4): This model contains four parameters (*t_0_*, *d*, *e*, F^*^) to be fitted. The *d* (<0) parameter is the sharpness of the response curve and *e* (>0) is the mid-response temperature. The rate of forcing (F^*^) is a sigmoid function that was in the range of (0–1).
  (4) $$Rf\left(x_{t}\right)=\{\begin{array}{c} 0\;if\;x_{t}<0\\ \frac{1}{1+e^{d\left(x_{t}-e\right)}}\;if\;x_{t}\geq 0 \end{array}$$

### 4.3. Models Parameterization and Validation

The model parameters were optimized using the Phenological Modeling Platform (PMP) software (version 5.5) [95]. The PMP is a platform that allows the users to build, adjust, simulate, test and validate phenological models intuitively and easily. It allowed us to use models included in the platform and to fit the model to our data, or to create a new model using the functions of the software. Each model is defined using meteorological and phenological data to estimate the most suitable model parameters, for a specific location and a predefined period of time. The software is very adaptable, i.e., allowing set the start date (t_0_) of the first phase of the model and the start date of each phase (constant or variable). 

The optimization of the parameter estimated by the PMP was carried out using the Metropolis simulated annealing algorithm [96] and following an interactive optimization procedure.

A multistep parameterization methodology was applied to estimate the timing of budburst and flowering using the dataset of four varieties independently (12 years per variety).

First, we tested whether any differences in the model estimation were observed, particularly at the start of accumulation of forcing units (*t_0_* date) considering the beginning of the dataset from September 1st of the previous year and from January 1st of the current year. This procedure allowed us to select the best data frame for each phenophase and variety (since, particularly for budburst, heat accumulation could start during December [31,68], allowing a first set of intermediate space for the parameter values to be imposed in the subsequent optimization of the models (Table 1)).

Second, a model calibration phase was conducted using each variety dataset independently, where the intermediate-fitted parameters for each model formulation (GDD model: t_b_; GDD Triangular model: *Tmin*, *Topt* and *Tmax*; the UniFORC model: *e*) were freely-adjusted. This procedure allowed us to achieve an optimized model with fixed parameters for future prediction of the budburst and flowering dates at the variety level.

Afterwards, the parameter set combination of the model providing the highest-performance for each variety was externally validated with the dataset of the other three varieties to evaluate the prediction accuracy of the estimated models. This fact allowed us to establish one overall unique model for budburst and flowering for all varieties. Finally, the performance of this model was compared with the attained considering all varieties in one dataset (12 years x 4 varieties, giving a total number of observations of 48).

The estimation, validation and selection of the best performing models for each variety and phenological phase were conducted taking into account 3 goodness-of-fit metrics: the model with the highest efficiency (EFF, Equation (5)), the lowest root mean square error (RMSE, Equation (6)) and the mean absolute deviation (MAD, Equation (7)) between observed and predicted values. The following equations were applied:

(5) $$EFF=\frac{\left(SStot-SSres\right)}{SStot}$$

(6) $$RMSE=\sqrt{\frac{SSres}{n}}$$

(7) $$MAD=\frac{\sum_{i01}^{n}\left|Xobs_{i}-Xpre_{i}\right|}{n}$$

where *SStot* is the Total Sum of Squares, *SSres* is the Residual Sum of Squares, *n* the number of observations and *Xobsi* and *Xprei* are, respectively, the observed values and predicted values.

For the best-fitted models’ results presentation, an analysis of the frequencies of the differences between the expected and observed dates for each phenophase was applied. Also, a linear regression through the origin was conducted between the predicted and observed dates of budburst and flowering. If the concordance line of this regression passed through the origin and it had a regression coefficient of unity or was very close to one, this would show that there are no significant differences between the budburst and flowering dates predicted and observed, marking the lack of bias.

## 5. Conclusions

This study provides relevant information for forecasting and predicting the temporal evolution of grapevines in the face of climate change. It is important to have more regional forecasting models to assess the effect of climate change more assertively on the bioclimatic conditions of a given wine region. With the modeling approach used in our study, it was possible to achieve a global prediction model for the four studied varieties and also to obtain data on the adaptability of the different varieties by providing their optimal base temperatures. Although we should test this model with more varieties to increase the robustness and significance of the results, it lays the foundation to be able to employ this type of approach.

## Figures and Tables

**Table 1:** Results of the estimation process for the three models studied for the Budburst and Flowering phenological phases. DOY: day of the year, Tb: base temperature, F: fitted, (M, m): maximum and minimum, maxT: maximum temperature, minT: minimum temperature, d: defines the sharpness of the response curve and e: mid-response temperature. The bold letters highlight the best performing models for each variety and phenological phase.

		Budburst				Flowering			
**Growing Degree Days (GDD)**									
		DOY	Tb			DOY	Tb		
		F (M,m)	F (M,m)			F (M,m)	F (M,m)		
**Albariño**		62 (70; 62)	8 (9; 8)			27 (29; 27)	7 (7; 8)		
	**EFF**	0.825 (0.825; 0.800)				0.929 (0.929; 0.919)			
	**RMSE**	3.809 (4.079; 3.809)				2.492 (2.661; 2.492)			
	**∑Heat**	75.141 (78.969; 36.888)				533 (590; 453)			
**Godello**		49 (49; 48)	5 (5; 5)			**52 (53; −261)**	**6 (6; 25)**		
	**EFF**	0.854 (0.854; 0.851)				**0.965 (0.965; −34.576)**			
	**RMSE**	2.791 (2.826; 2.791)				**1.494 (47.869; 1.494)**			
	**∑Heat**	208.931 (229.050; 194.852)				**626.092 (668.958; 22.492)**			
**Loureira**		79 (79; -236)	10 (18; 8)			72 (71; −287)	6 (24; 6)		
	**EFF**	0.810 (0.810; −55.213)				0.911 (0.911; −42.230)			
	**RMSE**	3.327 (57.250; 3.327)				2.250 (49.700; 2.250)			
	**∑Heat**	18.616 (383.187; 18.077)				587.035 (611.629; 30.320)			
**Treixadura**		**49 (49; 48)**	**5(5; 5)**			52 (54; 52)	7 (7; 6)		
	**EFF**	**0.883 (0.883; 0.880)**				0.949 (0.949; 0.946)			
	**RMSE**	**2.319 (2.342; 2.319)**				1.982 (2.052; 1.982)			
	**∑Heat**	**228.503 (248.592; 217.663)**				575.441 (622.268; 549.083)			
**Growing Degree Days (GDD) Triangular**									
		DOY	Tb	minT	maxT	DOY	Tb	minT	maxT
**Albariño**		70 (71; 62)	13 (28; 13)	9 (9; 8)	24 (49; 20)	27 (30; 23)	27 (30; 22)	7 (9; 7)	32 (50; 30)
	**EFF**	0.852 (0.852; 0,787)				0.929 (0.929; 0.913)			
	**RMSE**	3.502 (4.203; 3.502)				2.564 (2.759; 2.488)			
	**∑Heat**	7.381 (10.113; 2.371)				26 (37; 22)			
**Godello**		49 (50; 42)	16 (29; 15)	5 (6; 4)	50 (50; 20)	**52 (56; 50)**	**23 (29; 22)**	**6 (7; 5)**	**31 (50; 28)**
	**EFF**	0.854 (0.854; 0.828)				**0.966 (0.966; 0.958)**			
	**RMSE**	2.788 (3.033; 2.788)				**1.474 (1.653; 1.474)**			
	**∑Heat**	18.528 (20.567; 8.344)				**37.146 (38.823; 26.154)**			
**Loureira**		79 (80; 71)	14 (21; 12)	10 (10; 8)	29 (50; 20)	70 (72; 65)	26 (29; 22)	6 (7; 5)	32 (50; 31)
	**EFF**	0.834 (0.834; 0.784)				0.913 (0.913; 0.900)			
	**RMSE**	3.111 (3.549; 3.111)				2.233 (2.392; 2.233)			
	**∑Heat**	5.743 (11.446; 3.618)				30.304 (35.534; 22.976)			
**Treixadura**		**52 (64; 51)**	**23 (29; 23)**	**7 (8; 6)**	**26 (42; 25)**	52 (56; 50)	24 (30; 22)	7 (7; 6)	25 (48;25)
	**EFF**	**0.954 (0.954; 0.936)**				0.954 (0.954; 0.940)			
	**RMSE**	**1.890 (2.230; 1.890)**				1.889 (2.153; 1.889)			
	**∑Heat**	**35.587 (35.587; 25.130)**				34.216 (39.633; 26.519)			
**UniFORC**									
		DOY	d		e	DOY	d		e
**Albariño**		70 (72; 70)	−19.326 (−2.969; −40.000)		11.538 (11.538; 10.893)	11 (8;70)	−0.937 (−0.237; −35.849)		12.772 (19.074; 11.658)
	**EFF**	0.857 (0.857; 0.789)				0.912 (0.912; 0.870)			
	**RMSE**	3.450 (4.183; 3.450)				2.768 (3.379; 2.768)			
	**∑Heat**	7.071 (8.701; 7.071)				45.930 (51.484; 21.405)			
**Godello**		34 (61; 34)	−0.265 (−0.221; −40.000)		15.507 (15.507; 8.807)	**28 (69; 28)**	**−0.217 (−0.150; −0.761)**		**19.490 (19.490; 10.521)**
	**EFF**	0.856 (0.856; 0.775)				**0.958 (0.958; 0.870)**			
	**RMSE**	2.778 (3.469; 2.778)				**1.648 (2.889; 1.648)**			
	**∑Heat**	11.012 (27.433; 11.012)				**22.696 (65.110; 22.696)**			
**Loureira**		80 (81; 74)	−40.000 (−3.336; −40.000)		12.164 (12.295; 10.996)	70 (74; 49)	−0.293 (−0.227; −1.455)		14.429 (18.564; 10.663)
	**EFF**	0.874 (0.874; 0.839)				0.900 (0.900; 0.801)			
	**RMSE**	2.715 (3.064; 2.715)				2.387 (3.368; 2.387)			
	**∑Heat**	5.464 (10.305; 5.459)				36.287 (61.146; 21.801)			
**Treixadura**		**72 (72; 70)**	**−0.923 (−0.923; −40.000)**		**10.811 (10.811; 9.668)**	26 (67; 8)	−0.281 (−0.243; −0.942)		17.393 (21.284; 11.573)
	**EFF**	**0.888 (0.909; 0.882)**				0.928 (0.927; 0.868)			
	**RMSE**	**2.273 (2.328; 2.046)**				2.379 (3.189; 2.379)			
	**∑Heat**	**11.376 (16.148; 11.376)**				26.634 (55.866; 14.876)			

**Table 2:** Goodness-of-fit indicators for the GDD model per each variety for estimation and validation process using the dataset for each other’s grapevine varieties. Fitted of t_0_: day of the year and Tb: base temperature. Bold letters show the best performing models for each variety and phenological phase.

	**Budburst**			
	**Albariño**	**Godello**	**Loureira**	**Treixadura**
	t_0_ = 62; Tb = 8	t_0_ = 49; Tb = 5	t_0_ = 79; Tb = 10	t_0_ **= 49; Tb = 5**
**Albariño**		EFF = 0.681 RMSE = 5.148	EFF = 0.657 RMSE = 5.334	EFF = 0.681 RMSE = 5.148
**Godello**	EFF = 0.733 RMSE = 3.775		EFF = 0.526 RMSE = 5.034	**EFF = 0.854 RMSE = 2.793**
**Loureira**	EFF = 0.683 RMSE = 4.297	EFF = 0.603 RMSE = 4.809		EFF = 0.603 RMSE = 4.809
**Treixadura**	**EFF = 0.737 RMSE = 3.479**	**EFF = 0.883 RMSE = 5.148**	**EFF = 0.731 RMSE = 3.520**	
	**Flowering**			
	**Albariño**	**Godello**	**Loureira**	**Treixadura**
	t_0_ = 27; Tb = 8	t_0_ **= 52; Tb = 6**	t_0_ = 71; Tb = 6	t_0_ = 52; Tb = 7
**Albariño**		EFF= 0.904 RMSE = 2.901	EFF= 0.827 RMSE = 3.890	EFF = 0.911 RMSE = 2.784
**Godello**	**EFF= 0.947 RMSE = 1.848**		**EFF = 0.915 RMSE = 2.339**	**EFF = 0.962 RMSE = 1.565**
**Loureira**	EFF= 0.769 RMSE = 3.634	EFF= 0.860 RMSE = 2.827		EFF = 0.842 RMSE = 3.004
**Treixadura**	EFF= 0.933 RMSE = 2.280	**EFF = 0.941 RMSE = 2.127**	EFF = 0.894 RMSE = 2.867	

**Table 3:** Phenological models selected to predict the dates of budburst and flowering. DOY: day of the year, Tb: base temperature, F: fitted, (M, m): maximum and minimum values attained.

Growing Degree Days (GDD)							
Budburst				Flowering			
		DOY	Tb			DOY	Tb
		F (M,m)	F (M,m)			F (M,m)	F (M,m)
**Treixadura**		**49** (49; 48)	5 (5; 5)	**Godello**		**52** (53; −261)	**6** (6; 25)
	**EFF**	0.883 (0.883; 0.880)			**EFF**	0.965 (0.965; −34.576)	
	**RMSE**	2.319 (2.342; 2.319)			**RMSE**	1.494 (47.869; 1.494)	
	**∑Heat**	228.503 (248.592; 217.663)			**∑Heat**	626.092 (668.958; 22.492)	

**Table 4:** Goodness-of-fit indicators of global GDD model for estimation process using the dataset for all grapevine varieties studied. Fitted t0: day of the year and Tb: base temperature.

	Budburst	Flowering
**Global Model**	**T0 = 63; Tb = 7**	**T0 = 52; Tb = 7**
	EFF = 0.731	EFF = 0.881
	RMSE = 4.153	RMSE = 2.979

## Data Availability

Data sharing is not applicable to this article.
